# A Controlled Clinical Study of Accelerated High-Dose Theta Burst Stimulation in Patients with Obsessive–Compulsive Disorder

**DOI:** 10.1155/2023/2741287

**Published:** 2023-12-07

**Authors:** Jin Jiang, Ke Wan, Yueling Liu, Yan Tang, Wenxin Tang, Jian Liu, Jiehua Ma, Chuang Xue, Lu Chen, Huichang Qian, Dandan Liu, Xinxin Shen, Ruijuan Fan, Yongguang Wang, Kai Wang, Gongjun Ji, Chunyan Zhu

**Affiliations:** ^1^School of Mental Health and Psychological Sciences, Anhui Medical University, Hefei, China; ^2^Collaborative Innovation Center of Neuropsychiatric Disorders and Mental Health, Hefei, China; ^3^Anhui Province Key Laboratory of Cognition and Neuropsychiatric Disorders, Hefei, China; ^4^Affiliated Mental Health Center & Hangzhou Seventh People's Hospital, Zhejiang University, School of Medicine, Hangzhou, China; ^5^Laboratory for Traumatic Stress Studies, CAS Key Laboratory of Mental Health, Institute of Psychology, Chinese Academy of Sciences, Beijing, China; ^6^Department of Psychology, University of Chinese Academy of Sciences, Beijing, China; ^7^Department of Psychology, Zhejiang Sci-Tech University, Hangzhou, China; ^8^Department of Neurology, The First Affiliated Hospital of Anhui Medical University, Anhui Medical University, Hefei, China; ^9^Institute of Artificial Intelligence, Hefei Comprehensive National Science Center, Hefei, China; ^10^Department of Psychology, The Second Affiliated Hospital of Anhui Medical University, Anhui Medical University, Hefei, China

## Abstract

**Background:**

Obsessive–compulsive disorder (OCD) is frequently treated using a combination of counseling, drugs, and, more recently various transcranial stimulation protocols, but all require several weeks to months for clinically significant improvement, so there is a need for treatments with faster onset. This study investigated whether an accelerated high-dose theta burst stimulation (ahTBS) protocol significantly improves the efficacy of OCD compared to traditional 1-Hz repetitive transcranial magnetic stimulation (rTMS) in the routine clinical setting.

**Method:**

Forty-five patients with OCD were randomized into two groups and treated with ahTBS or 1-Hz rTMS for 5 days. Patients were assessed at baseline at the end of treatment using the Yale–Brown Obsessive–Compulsive Scale (Y-BOCS).

**Results:**

After 5 days of treatment, there was a significant decrease in Y-BOCS scores in both groups (*p* < 0.001), and the difference between the two groups was not statistically significant (group × time interaction, *F* = 1.90, *p*=0.18). There was also no statistically significant difference in other secondary outcome indicators, including depression, anxiety symptoms, and response rate. However, the ahTBS group had a greater trend in response rate. Neuropsychological testing showed no negative cognitive side effects of either treatment.

**Conclusion:**

Accelerated high-dose TBS is as safe and has comparable short-term efficacy to traditional 1-Hz rTMS for the clinical treatment of OCD. Further research is needed to explore optimal ahTBS parameters, validate the utility of this treatment modality, and identify factors predictive of rapid clinical response to guide clinical decision-making. This trial is registered with NCT05221632.

## 1. Introduction

Obsessive–compulsive disorder (OCD) is a common psychiatric disorder afflicting 2.5%–3% of the general population [1]. It is characterized by current and intrusive thoughts or mental images (obsessions) and repetitive stereotypical or ritualistic behaviors (compulsions) that cause distress and interfere with daily function [1]. Currently recognized treatments for OCD include cognitive behavioral therapy (CBT) and selective serotonin reuptake inhibitors (SSRIs) [2]. However, medications and psychotherapies for OCD may require several weeks or months to produce clinically significant improvement. For instance, a meta-analysis [3] found that SSRIs showed significant benefit only after 2 weeks of treatment compared to placebo, while other studies have found that it takes at least 12 weeks of moderate-to-high-dose SSRI treatment to elicit a clinically significant response [2, 4]. To achieve rapid results in the clinical setting, transcranial magnetic stimulation (TMS) is often used in addition to medication as a therapeutic booster and accelerator [2, 5]. However, the traditional rTMS protocol usually requires 4–6 weeks [6–9], and some patients may be unable to meet the time requirements or tolerate such long courses of treatment.

A theta-burst stimulation (TBS) modality was recently introduced that takes less time and may have longer-lasting effects. This accelerated high-dose TBS (ahTBS) [10] protocol, which is delivered over only 5 consecutive days, demonstrated promising results among patients with refractory depression and is currently under study to address safety, tolerability, and efficacy for several other neuropsychiatric and neurological disorders. Sun et al. [11] found that ahTBS provided remarkable relief from freezing of gait in 91.7% of Parkinson's disease patients after 5 days of treatment without adverse events. Williams et al. [12] also applied ahTBS at the right frontal pole for 5 consecutive days to treat a small group of patients with refractory OCD and found a 57% response rate by the second week. Given the small sample size, however, further validation is needed.

The purpose of this study was to investigate whether optimizing the rTMS protocol in the real-world clinical setting can improve OCD treatment efficacy. In routine clinical practice, TMS is usually used in combination with medication. However, most previous studies exploring the efficacy of TMS were conducted in a strict laboratory setting while controlling the drug dose period prior to treatment [13–15]. The extent to which these findings can be generalized to the typical clinical situation is unclear. Therefore, to improve the ecological validity of the findings, this study optimized the sequence of TMS in routine clinical practice to explore whether the optimized ahTBS sequence could show superior efficacy to traditional 1-Hz rTMS. Based on the results of previous randomized controlled trials by our group [16] and a meta-analysis [17], the right presupplementary motor area (pre-SMA) was selected as the therapeutic target.

## 2. Materials and Methods

### 2.1. Standard Protocol Approval, Clinical Trial Registration, and Patient Consent

The study was conducted at the Seventh People's Hospital of Hangzhou, and the research plan was reviewed and approved by the Ethics Committee of the Seventh People's Hospital of Hangzhou. Prior to enrollment, all study candidates were given a full explanation of study methods, goals, the voluntary nature of participation, and the possible side effects of TMS, and all provided written informed consent in accordance with the Helsinki Declaration. The study protocol was also accepted for registration at ClinicalTrials.gov (Protocol ID: NCT05221632). The study protocol mentions that patients were assessed at weeks 2 and 4 post-treatment with Yale–Brown Obsessive–Compulsive Scale (Y-BOCS) follow-up. However, follow-up data were not included in the analyses of this study due to the high rate of patient dropout during the follow-up period. Analysis of Y-BOCS for patients who completed all follow-up visits is detailed in Figure S1.

### 2.2. Study Design

The sample size was estimated using G ^*∗*^ Power 3.1 software. In a previous study [18], the effect size (Cohen's *d* value) for the efficacy of cTBS on OCD was 0.44 (which converts to a partial eta squared value of 0.05). With a modest effect size in our study, a power of 95%, alpha of 0.05, two groups and two repeated measurements, the proposed study required 10 participants per group.

The participants were randomly divided into an ahTBS group and 1-Hz rTMS group by random number generation. Both groups received the indicated mode of TMS for 5 consecutive days. Raters were also blinded to group information and except for clinical assessment, did not take part in any other aspects of the study. All subjects were evaluated before and after treatment.

### 2.3. Inclusion and Exclusion Criteria

All study participants were patients with OCD hospitalized at the Seventh People's Hospital of Hangzhou. Inclusion criteria were as follows: (a) diagnosed by more than two clinical psychiatrists according to the diagnostic criteria of the International Statistical Classification of Diseases and Related Health Problems 10th Revision (ICD-10); (b) overall Y-BOCS score of 16 or higher, or subscale score of 10 points or higher and Hamilton Depression Scale (HAMD) score less than 17; (c) between 18 and 45 years of age with normal intelligence and able to cooperate with various examinations and assessments; (d) both patients and families agreed to participate in the study; and (e) a stable drug dose during the TMS treatment sessions.

Exclusion criteria were (a) other mental illnesses and severe medical diseases; (b) neurological disorders; (c) a history of substance abuse and drug dependence; (d) increased intracranial pressure due to infarcts or trauma; (e) neurostimulation such as TMS, transcranial direct current stimulation (tDCS), or electroconvulsive therapy in the past 6 months; (f) immovable metal objects around or inside the head or other contraindications to magnetic resonance imaging; and (g) pregnant or lactating.

### 2.4. Treatment

The resting motor threshold (RMT) of each subject was determined before the first TMS treatment as the lowest stimulation intensity capable of inducing a motor response of at least 50 *μ*V at the relaxed abductor pollicis brevis muscle in at least 5 of 10 TMS pulses delivered to the contralateral primary motor cortex. Each participant also received both structural MRI and resting-state functional MRI scans. The personalized target location (right pre-SMA) was calculated using the TMS target [19] and SPM12 (https://www.fil.ion.ucl.ac.uk/spm). For detailed descriptions, refer to Ji et al. [16].

Subjects in the ahTBS group received 5 consecutive days of ahTBS to the right pre-SMA. Each modified continuous TBS (cTBSmod) session was comprised of 1,800 pulses delivered in a continuous train of 600 theta bursts. Each theta burst consisted of three pulses at 50 Hz, and bursts were repeated at 5 Hz. Ten sessions were applied per day at 50-min intersession intervals [10] (18,000 total pulses per day). Stimulation was delivered at 80% of the RMT. Subjects in the rTMS group received 1,800 TMS pulses at 1 Hz to the right pre-SMA each day for 5 consecutive days, also at 80% of RMT.

### 2.5. Assessments

Two trained psychology graduate students administered clinical and neuropsychological assessments. Clinical evaluations included the Y-BOCS, Obsessive–Compulsive Inventory-Revised (OCI-R), HAMD, and Hamilton Anxiety Scale (HAMA), while neuropsychological tests included the trail making test (TMT), Stroop color word test (SCWT), and digit span (DS) test.

Tests were conducted at baseline and again after 5 days of treatment. The primary outcome measure was the Y-BOCS, which is the gold standard for assessing the severity of OCD symptoms [20]. The scale consists of 10 items divided into two subscales, obsessive thinking and compulsive behavior, and each item is scored on a scale from 0 to 4 according to symptom severity and frequency of occurrence, with a higher score indicating more severe symptoms. Treatment response was defined as a 35% or greater decrease in Y-BOCS score relative to baseline.

Throughout the course of TMS treatment, all participants underwent rigorous monitoring of adverse effects, including the use of self-reporting to record side effects after each treatment session.

### 2.6. Statistical Analyses

All statistical analyses were conducted using SPSS version 21.0. Categorical variables were compared between groups by *χ*^2^ or Fisher's exact test as indicated, while continuous variables were compared by independent samples *t*-test. Drug equivalents were compared between groups by the Mann–Whitney *U*-test. Assessment scores, including the primary outcome variable (Y-BOCS score) and other secondary outcome variables, were compared by repeated measures analysis of variance (ANOVA) with assessment time (pre-TMS and post-TMS) as a within-group factor and TMS protocol (rTMS or ahTBS) as the between-group factor. All statistical tests were two-sided, with *p* < 0.05 regarded as statistically significant.

## 3. Results

### 3.1. Clinical and Demographic Characteristics of Study Participants

**Figure 1:**
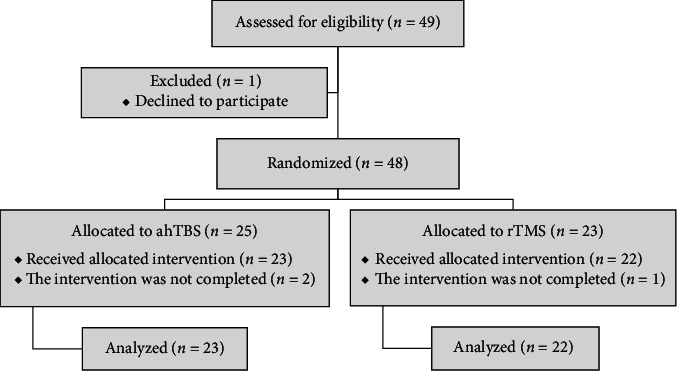
Flowchart of patient selection, grouping, and entry into analyses.

A total of 48 patients with OCD were randomized to receive either ahTBS or 1-Hz rTMS treatment. There were two dropouts in the ahTBS group and one in the 1-Hz rTMS group during treatment, so ultimately, data from 45 patients were included in the analysis (Figure 1).

All medications remained the same for the duration of treatment. All antidepressants (SSRIs and clomipramine) were converted to fluoxetine equivalents [21], all antipsychotics to chlorpromazine equivalents [22], and all sedatives to diazepam equivalents for analysis [23]. Fluoxetine equivalents did not differ significantly between ahTBS and 1-Hz rTMS groups (Mean Rank, 22.89 vs. 23.11, *U* = 250.50, *Z* = −0.06, *p*=0.96 by Mann–Whitney *U*-test). Similarly, there was no group difference in chlorpromazine equivalents (mean rank, 24.57 vs. 21.36, *U* = 217.00, *Z* = −0.88, *p*=0.38) and no significant difference in diazepam equivalent (19.52 vs. 26.64, *U* = 173.00, *Z* = −1.88, *p*=0.06).

There was no significant difference in baseline demographic variables, other clinical metrics, or neuropsychological test scores between groups (Table 1).

### 3.2. Symptom Scale and Neuropsychological Test Outcomes

**Figure 2:**
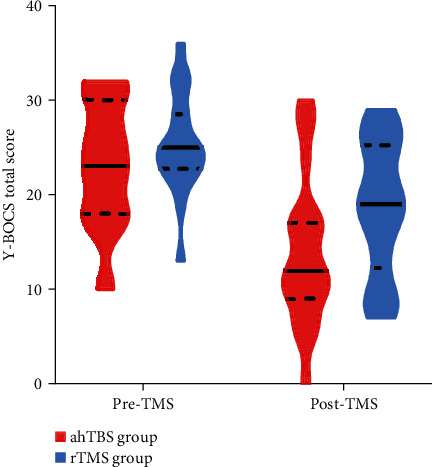
Y-BOCS scores in ahTBS and rTMS groups.

**Figure 3:**
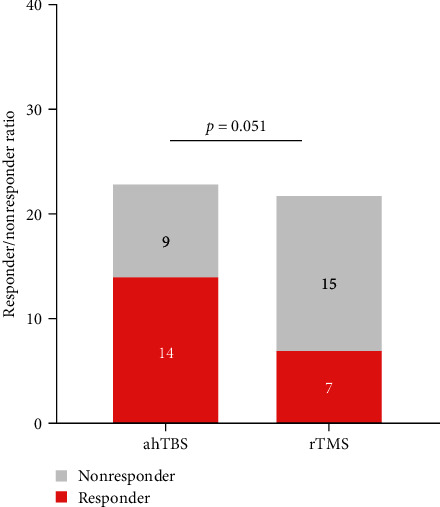
Y-BOCS decreased more than 35% (responder) in ahTBS and rTMS groups.

Scores on the Y-BOCS were significantly reduced in both groups following treatment (*p* < 0.001; Figure 2), and there was no significant time (pre-TBS vs. post-TBS) × group (ahTBS vs. 1-Hz-rTMS) interaction effect (*F* = 1.90, *p*=0.18; Table 2, Figure 2). There was also no significant difference in the Y-BOCS score percentage change following treatment between ahTBS and 1-Hz rTMS groups (mean = 39%, SD = 0.27 vs. mean = 27%, SD = 0.23, *t* = 1.60, *p*=0.12). However, there was a trend for a higher proportion of responders in the ahTBS group than the 1-Hz rTMS as defined by a 35% reduction in Y-BOCS score post-treatment (14 of 23 patients vs. 7 of 22, *χ*^2^ = 3.81, *p*=0.051; Figure 3).

**Figure 4:**
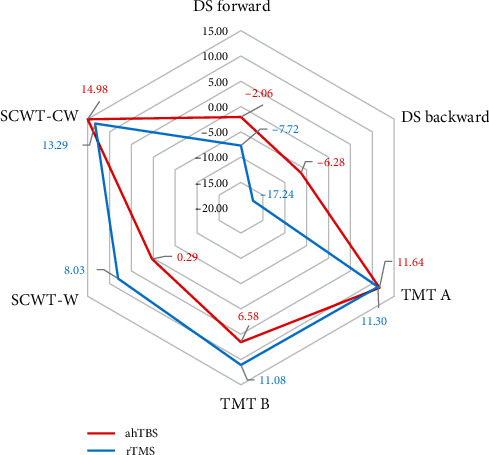
Neuropsychological tests for improved values-(pre–post)/pre × 100.

Repeated measures ANOVA revealed significant main effects of time (post-treatment vs. pretreatment) on OCI-R scores, HAMA scores, and HAMD scores (all *p* < 0.001), but no significant group × time interaction effects (HAMD: *F* = 0.02, *p*=0.89; HAMA: *F* = 0.26, *p*=0.62; OCI-R: *F* = 0.08, *p*=0.78). There were also no significant group × time interaction effects on TMT(B-A) (*F* = 0.13, *p*=0.72), SCWT (CW-W) (*F* = 0.91, *p*=0.35), DS forward (*F* = 3.00, *p*=0.09), and DS backward (*F* = 2.63, *p*=0.11) (Table 2), and no significant group difference in the percentage change for any of these neuropsychological tests (Figure 4).

### 3.3. Safety and Side Effects

No serious adverse events occurred during treatment. Only one patient in the ahTBS group reported a mild headache that resolved with rest, while the remaining patients were free of adverse events and seizures.

## 4. Discussion

This is the first study to compare the efficacy of accelerated high-dose TBS to the more widely used 1-Hz rTMS for the treatment of OCD in a routine clinical setting. After 5 days of treatment, Y-BOCS scores were significantly reduced in both groups, with no difference in the mean percentage drop. This suggested that ahTBS was comparable in efficacy to traditional 1-Hz rTMS for OCD. Notably, however, there was a trend for a greater response rate after 5 days of ahTBS. Moreover, ahTBS was well-tolerated, suggesting that this modality was a safe and effective alternative for the rapid treatment of OCD symptoms.

Under ideal conditions, treatments are deemed effective by randomized controlled trials (RCTs), while “real-world” research is typically observational and aims to measure the effectiveness of interventions in nonexperimental scenarios such as routine clinical practice [24]. This study was based on a rigorous RCT experimental design, with randomization of participants into groups and blinding of raters, but was conducted in routine clinical practice. We tried to improve the effectiveness of TMS by using the new sequences in routine clinical practice. According to one of the earliest studies, ahTBS demonstrated significant efficacy in patients with depression who do not respond to pharmacotherapy and a range of treatments [10], so ahTBS sequence may be a direction to optimize TMS.

To our knowledge, only one preliminary study has examined the efficacy of ahTBS in patients with OCD. The authors reported a remission rate of 57% in seven patients after 5 days of treatment [12], similar to the remission rate achieved in the current study. In the present study, however, the sample size was larger, and a group receiving the traditional 1-Hz rTMS was included for comparison. The 1-Hz rTMS protocol is currently the most frequently used TMS treatment for OCD [20, 25, 26], and previous studies have demonstrated significant reductions in Y-BOCS scores when applied to the pre-SMA [6, 7, 27]. We found that the overall reduction in symptom severity produced by ahTBS as measured by the average Y-BOCS score reduction was comparable to that produced by the traditional 1-Hz sequence, but there were more responders (defined by a 35% reduction in Y-BOCS score) in the ahTBS group. However, the difference did not reach significance. Larger scale studies are needed to resolve this uncertainty.

There are several possible explanations for why ahTBS was not clearly more efficacious than 1-Hz rTMS.

1. Despite the accelerated stimulation schedule used for ahTBS (10 daily treatments), a 5-day treatment cycle may still be insufficient to detect a significant difference in efficacy compared to 1-Hz TMS. For instance, TMS-induced neural plasticity may be a slower process [28].
2. Almost all patients were taking medications that could potentially interfere with the effects of TMS, including TBS-induced plasticity [29, 30]. Despite no significant differences in equivalent doses between treatment groups, medications may still have differentially influenced symptoms. If the study were limited to patients with drug-refractory OCD, perhaps ahTBS would have shown superior efficacy compared to 1-Hz TMS.
3. While the same high-dose sequence (18,000 pulses per day and a total dose of 90,000 pulses) achieved significant efficacy in patients with depression [10] and Parkinson's disease [11], this stimulation pattern may not be as effective in patients with OCD. Indeed, there may be substantial individual and group differences in the response to noninvasive brain stimulation protocols [31]. In addition, a recent study found that doubling the stimulation duration of TBS may result in a reversal of effect with regard to cortical excitability [32], so the use of high doses also requires careful consideration, and higher doses may not necessarily enhance the neurological response.
4. Stimulation intensity is an important factor influencing cTBS therapeutic efficacy [33] and after-effects [34]. In the aforementioned foundational studies [10], ahTBS was delivered at 90% of RMT, while 80% of RMT was chosen here for safety considerations. However, it was possible that 80% of RMT was insufficient for therapeutic effects. While there is currently no clear evidence that high-intensity TBS is more effective for treating OCD, further research is needed to determine the optimal stimulation intensity for OCD patients.

## 5. Conclusion

In conclusion, ahTBS was as safe and had comparable short-term efficacy to traditional 1-Hz rTMS for the treatment of OCD in routine clinical practice. While this study had high ecological validity, future research is needed to explore optimal parameters for ahTBS.

## Figures and Tables

**Table 1:** Baseline demographic and clinical characteristics of the participants.

Measures	ahTBS (*n* = 23)	rTMS (*n* = 22)	*t/χ* ^2^	*p*
	Mean ± SD	Mean ± SD		
Sex (male/female)	16/7	10/12	2.68	0.10^b^
Age (years)	26.61 (6.99)	27.77 (7.50)	−0.54^a^	0.59
Education (years)	13.43 (2.17)	13.82 (2.75)	−0.52^a^	0.61
Duration of illness (years)	4.48 (4.91)	3.18 (4.82)	0.89^a^	0.38
*Symptom estimations*				
Y-BOCS total	23.13 (6.68)	25.41 (5.12)	−1.28^a^	0.21
Y-BOCS obsession	13.13 (2.62)	13.14 (2.93)	−0.01^a^	0.99
Y-BOCS compulsion	10.00 (5.40)	12.27 (3.56)	−1.67^a^	0.10
OCI-R	23.09 (14.53)	23.68 (11.77)	−0.15^a^	0.88
HAMD	8.22 (4.63)	7.59 (5.31)	0.42^a^	0.68
HAMA	7.91 (5.61)	6.95 (5.10)	0.60^a^	0.55
*Neuropsychological tests*				
TMT (B-A)	49.89 (39.67)	39.63 (16.29)	1.13^a^	0.27
SCWT (s)	12.69 (5.73)	11.12 (6.73)	0.84^c^	0.41^c^
DS forward	9.43 (1.65)	8.77 (0.97)	1.63^a^	0.11
DS backward	6.52 (1.59)	6.73 (1.64)	−0.43^a^	0.67
*Medication status*	Mean rank	Mean rank	*Z*	*p*
Fluoxetine equivalent (mg)	22.89	23.11	−0.06	0.96
Chlorpromazine equivalent (mg)	24.57	21.36	−0.88	0.38
Diazepam equivalent (mg)	19.52	26.64	−1.88	0.06

*Note*: Data are presented as mean (SD). Y-BOCS: Yale–Brown Obsessive–Compulsive Scale; HAMD: Hamilton Depression Scale; HAMA: Hamilton Anxiety Scale; OCI-R: obsessive–compulsive inventory-revised; TMT: trail making test; SCWT, Stroop color word test; DS: digit span. All antidepressant doses were converted to fluoxetine equivalents, all antipsychotic doses to chlorpromazine equivalents, and all sedative drug doses to diazepam equivalents. ^a^Paired *t*-test. ^b^Fisher's exact test. ^c^One patient in the ahTBS group did not take the test.

**Table 2:** Outcome comparison between ahTBS and rTMS treatment groups.

Measures	ahTBS (*n* = 23)		rTMS (*n* = 22)		ANOVA time effect (*p*)	ANOVA group × time interaction
	Pre-TMS	Post-TMS	Pre-TMS	Post-TMS	*p*	*p*
*Symptom estimations*						
Y-BOCS total	23.13 (6.68)	13.96 (7.76)	25.41 (5.12)	18.68 (7.13)	<0.001	0.18^a^
Y-BOCS obsession	13.13 (2.62)	8.09 (3.85)	13.14 (2.93)	9.77 (3.59)	<0.001	0.10^a^
Y-BOCS compulsion	10.00 (5.40)	5.87 (5.10)	12.27 (3.56)	8.91 (4.39)	<0.001	0.47^a^
OCI-R	23.09 (14.53)	14.78 (13.02)	23.68 (11.77)	16.09 (8.85)	<0.001	0.78^a^
HAMD	8.22 (4.63)	4.87 (4.37)	7.59 (5.31)	4.36 (4.44)	<0.001	0.89^a^
HAMA	7.91 (5.61)	4.61 (4.91)	6.95 (5.10)	4.09 (3.92)	<0.001	0.62^a^
*Neuropsychological tests*						
TMT (B-A)	49.89 (39.67)	43.37 (13.40)	39.6 (16.29)	36.43 (14.68)	0.29	0.72^a^
SCWT (CW-W)	12.69 (5.73)	7.83 (5.90)	11.12 (6.73)	8.26 (4.50)	<0.001	0.35^b^
DS forward	9.43 (1.65)	9.61 (1.73)	8.77 (0.97)	9.41 (1.10)	<0.001	0.09^a^
DS backward	6.52 (1.59)	6.83 (1.50)	6.73 (1.64)	7.59 (1.26)	<0.001	0.11^a^

*Notes*: Data are presented as mean (SD). Y-BOCS: Yale–Brown Obsessive–Compulsive Scale; HAMD: Hamilton Depression Scale; HAMA: Hamilton Anxiety Scale; OCI-R: obsessive–compulsive inventory-revised; TMT: trail making test; SCWT, Stroop color word test; DS: digit span. ^a^Repeated the measurement of ANOVA. ^b^One patient in the ahTBS group did not take the test.

## Data Availability

The experiment data are available from the corresponding author upon reasonable request.

## Additional Points

*Limitations and Prospects*. First, this was a single-center study with a small sample size, so the clinical efficacy of ahTBS needs to be further validated in a larger-scale multicenter study. Second, the intermediate and longer-term efficacy was uncertain as we did not conduct extensive follow-up due to patient drop-out. However, previous studies have shown that the clinical effects of ahTBS last at least 4–5 weeks post-treatment [10, 12], which may be longer than those of brief 1-Hz rTMS. Future studies with longer-term follow-up are warranted to compared the post-treatment efficacy of ahTBS to 1-Hz rTMS. Third, we may also consider the inclusion of a sham stimulation group to help distinguish the effects of drugs and to observe the effects of TMS more visually. Finally, in addition to clinical evaluation, neuroimaging, and neurophysiological techniques should be employed in further studies to identify the neural responses underlying the clinical effects of ahTBS.

## Supplementary Materials

Supplementary Materials Figure S1: Y-BOCS scores in ahTBS (*n* = 10) and rTMS (*n* = 15) groups.
