# Supplementary material for: A Controlled Clinical Study of Accelerated High-Dose Theta Burst Stimulation in Patients with Obsessive–Compulsive Disorder
Source: Neural Plast. 2023 Dec 7;2023:2741287. doi: 10.1155/2023/2741287 (PMC10721349; doi:10.1155/2023/2741287)
Supplement: Supplementary Materials — Figure S1: Y-BOCS scores in ahTBS (n = 10) and rTMS (n = 15) groups. [file 2741287.f1.docx]

# Supplementary Material

Patients were assessed for Y-BOCS at Pre-TMS, Post-TMS treatment (after 5 days of treatment), 2-week follow-up (two weeks after treatment,) and 4-week follow-up (four weeks after treatment). Ultimately, there were 10 in the ahTBS group and 15 in the rTMS group who completed the 2-week and 4-week follow-ups. There was no significant difference in baseline demographic variables and other clinical metrics between groups. The results of the repeated measures ANOVA showed the evolution of Y-BOCS scores (Figure. S1) was no significant group × time interaction effects in the two groups between Pre-TMS, Post-TMS, 2-week and 4-week (F=0.13, p=0.91).

Figure S1. Y-BOCS scores in ahTBS (n=10) and rTMS (n=15) groups.
